# Iodine Supplementation in the Newborn

**DOI:** 10.3390/nu6010382

**Published:** 2014-01-20

**Authors:** Paolo Ghirri, Sara Lunardi, Antonio Boldrini

**Affiliations:** Department of Clinical and Experimental Medicine, Neonatal Intensive Care and Neonatology Unit, via Roma 55, Pisa 56126, Italy; E-Mails: saralunardi@hotmail.it (S.L.); antonio.boldrini@med.unipi.it (A.B.)

**Keywords:** iodine deficiency, iodine supplementation, thyroid function, newborn

## Abstract

Iodine deficiency can be defined as the world’s greatest single cause of preventable brain damage. Fetal and neonatal hypothyroidism, caused by iodine deficiency can be prevented prior to conception and then during pregnancy and lactation when an adequate iodine supplementation is ensured. Extremely low birth weight preterm babies risk having a negative iodine balance status in the first weeks of life, exacerbating the hypothyroxinaemia of the prematurity. It is important to ensure that these babies are provided with an adequate iodine intake from the first days of life. Mothers and newborns should avoid environmental iodine excess during pregnancy or lactation.

## 1. Introduction

Traces of iodine are present in food and water in variable amounts, depending on a number of factors. Its concentration in sea-water as iodide is about 50 µg/L. Iodide in seawater is oxidized and volatilizes back into the soil through rain. Nevertheless, in mountainous areas or regions where frequent flooding occur the iodine water-soil cycle is slower, and water and the soil are deprived of iodine [1]. Iodine content in food is variable as it is affected by soil, irrigation, and fertilizers. Seafood usually has a higher iodine content. Iodine has a major role within the human body: it is crucial in the production of the thyroid hormone. It is absorbed by the stomach and the duodenum and then cleared from the blood by the thyroid; before being absorbed, it is converted into iodide ion.

When the iodine intake is sufficient, the thyroid clears up about the 10% of the overall element circulating in blood, whilst in chronic iodine deficiencies this percentage can exceed 80% [1,2]. The active transport of iodine to the thyroid is mediated by the Sodium-Iodine Symporter (NIS) and regulated both by the Thyroid Stimulating Hormone and the iodine concentration in blood. Iodine is then covalently bound to tyrosine residues in thyroglobulin molecules, forming monoiodotyrosine (MIT) and diiodotyrosine (DIT). Thyroid Hormones Tri-iodo-thyronine (T3) and Thyroxine (T4) originate from the combination of MIT and DIT after having been separated from thyroglobulin. Circulating T4 (the most common thyroid hormone) originates entirely from the thyroid, while T3, the active form of thyroid hormone, originates mostly from the peripheral deiodination of T4 [3]. Cleaved by the peripheral deiodinases the iodine of the thyroid hormones re-enters circulation so that it can be reused by the thyroid. The iodine in excess is cleared from the body mainly through urine (90%) and on small amounts excreted through feces or sweat.

Iodine deficiency can be defined as the world’s greatest single cause of preventable brain damage. Currently, two billion people worldwide have insufficient iodine intake and 31.5% of children in school age have insufficient iodine intake [4]. Several European countries as well are still suffering from mild iodine deficiency [5]. This statistics are the drive behind a worldwide campaign to assure an adequate supplementation.

The main strategy to enhance iodine supplementation so far is the Universal Salt Iodization: according to WHO (*World Health Organization*), UNICEF (*United Nations International Children’s Emergency Fund*) and ICCIDD (*International Council for the Control of Iodine Deficiency Disorders)* iodine should be added at a level of 20–40 mg on every kg of salt [1]. Other strategies include bread iodization, drinking or irrigation water supplementation, or animal-fodder fortification; nevertheless salt iodization is recommended as salt is virtually consumed by everyone throughout the year and iodization technology is relatively easy. Important as it is in the synthesis of thyroid hormones, iodine plays a major role in the early growth and development of many organs, particularly the brain and the central nervous system, and in many metabolic pathways [3]. Consequently, iodine insufficiency, occurring when iodine intake falls below recommended levels and the thyroid is no longer able to produce enough thyroid hormones, can impair neurodevelopment [3]. When the maternal iodine status is insufficient the fetus suffers both from lack of the maternal T4, in a period during which the fetal hypothalamic axis is not fully functioning yet, and from impaired fetal hormones production as a consequence to the limited fruition of placental iodine. Iodine deficiency is a major threat during pregnancy and early childhood as these periods are critical in the development of the neural system of the fetus and the child.

Disorders caused by severe iodine deficiency before or during pregnancy range from decreased fertility to trophoblastic or embryonic damage, miscarriage, stillbirth or increased infant mortality, cretinism, congenital abnormalities, and psychomotor defects. Neonatal iodine deficiency causes neonatal goiter and can lead to moderate to severe hypothyroidism [3]. Two different forms of severe neonatal hypothyroidism were recognized in the original description of cretinism [1]: neurological cretinism, which consists in its most acute form of severe mental retardation, squint, deafness, and motor spasticity; and myxedematous cretinism that leads to severe growth retardation with short stature and the typical features of hypothyroidism (but less severe mental retardation) [1]. It is likely that neurological cretinism is related to maternal iodine deficiency in the early period of pregnancy while the myxedematous form seems to be related to a lack of iodine in the late pregnancy stages or in the first months of life [6].

Goiter, hypothyroidism, and impaired cognitive function can also occur during childhood or adolescence if iodine supplementation is not sufficient [7].

## 2. Assessing Iodine Deficiency

Urinary iodine (UI) is a sensible marker of the recent iodine intake. Iodine in excess is mostly excreted in urine. Even if the iodine urinary excretion in an individual varies on a daily basis, its concentration can be a reliable marker of the recent iodine intake when considering a statistically significant number of samples for a certain population. Iodine deficiency is considered to be a major problem when the median UI falls below 100 µg/L [1,4,8] (Table 1).

**Table 1:** Urinary iodine concentration and iodine intake [1,4,8].

Iodine Intake	Median Urinary iodine Concentration				
	General Population	Pregnant Women	Lactating Women	Children < 2 Years Old	School-Aged Children
Insufficient	<100 µg/L	<150 µg/L	<100 µg/L	<100 µg/L	<100 µg/L
Adequate	100–199 µg/L	150–249 µg/L	≥100 µg/L	≥100 µg/L	100–199 µg/L
More than adequate	200–299 µg/L	250–499 µg/L	-	-	200–299 µg/L
Excessive	>300 µg/L	≥500 µg/L	-	>180 µg/L	>300 µg/L

Even if UI is a reliable marker of the iodine status in a certain population, it cannot be used for individual diagnosis and treatment. It would be necessary to collect repeated urine samples from an individual over a period of time in order to correctly evaluate its iodine status [8]. It can be challenging to correctly identify the iodine status in a patient during pregnancy or lactation [9]. Iodine is concentrated in the mammary glands, hence the UI can lead to underestimate iodine intake.

Goiter rate, serum TSH, or Tg levels as well are useful assessments to determine the iodine status in a population, while the measurement of thyroid hormones remains a fairly unprecise methodology for this endeavor. The UI is a marker for recent iodine intake, whilst goiter rate marks a long-term response to iodine deficiency. The latter can be evaluated via neck inspection and palpation or through ultrasound scan and it can identify the severity of iodine deficiency in a population. We identify iodine-sufficiency when the goiter rate is below the 5%. Goiter rates ranging from 5% to 19.8% identify mild deficiency areas, moderately deficient areas ranges between 20% and 29.9% while severely deficient regions have goiter rates pushing above 30% [1].

TSH could be used as a sensible marker in the neonatal period (it seems to be a reliable indicator of even marginal deficiency when measured at three to four days of age) while it is a relatively insensitive indicator in older children or in adults. The World Health Organization (WHO) has suggested that, when a sensitive Assay is used on samples collected three to four days after birth, a <3% frequency of TSH concentrations >5 mIU/L indicates iodine sufficiency in a population [10]. Thyroid hormones usually show a variably increased TSH with lower serum T4 and normal/high levels of T3.

Tg is a sensitive indicator of both low and excess iodine intake (a median Tg that is lower than 13 μg/L indicates iodine sufficiency in a population) [11]. Crucially, it is also an early marker of iodine repletion (it falls quickly after iodine supplementation has started); there is, nevertheless, a risk of underestimation when anti-Tg antibodies are present.

It is important to remember that during pregnancy a small decrease in the concentration of serum free T4, a moderate increase of thyroglobulin (Tg) levels or an increase in TSH or Tg levels in cord blood samples are not signs of iodine deficiency.

## 3. Recommended Dosages of Daily Supplementation According to Age

Several studies have tried to estimate iodine requirements. In 2001, the U.S. Institute of Medicine (IOM) published its recommendation regarding iodine intake using three different categories of dietary reference intakes. The three categories are: Estimated Average Requirements (EAR), which indicates the daily intake necessary to meet the criterion of adequacy for a certain group of people (the nutrient intake of a population is satisfactory when 97%–98% of individuals meet the EAR [12]), Adequate Intake (AI), which is obtained by experimental observation and used when there is not enough scientific evidence to calculate an EAR, and Recommended Daily Allowance (RDA), which is the average daily intake sufficient to provide the gender and age target requirements in nearly all individuals and is defined as the EAR plus twice the coefficient of variation in the population (or as the 140% of the EAR) [13] (see Table 2).

**Table 2:** Recommended iodine intakes.

Source	Women of Reproductive Age (µg/day)	Pregnant Women (µg/day)	Lactating Women (µg/day)	Newborns and Infants (µg/day)	School Aged Children (µg/day)
Institute of Medicine (IOM), 2001	95 ** 150 °	160 ** 220 °	209 ** 290 °	110 (0–6 months) * 130 (7–12 months) * 65 (1–3 years) ** 90 (1–3 years) °	65 (4–8 years) ** 73 (9–13 years) ** 95 (14–18 years) ** 90 (4–8 years) ° 120 (9–13 years) ° 150 (14–18 years) °
Delange, 2004	-	250–300	225–350	90	-
WHO, 2007	150	250	250	90	120 (6–12 years)

* Adequate Intake (AI); ** Estimated Average Requirements (EAR); ° Recommended Dietary Allowances (RDAs) (140% of the EAR).

In 2004, Delange [14] suggested his recommended iodine dosage after a critical review of the relevant literature. The doses he suggested were 250–300 µg/day during pregnancy, 225–350 µg/day during lactation, and 90 µg/day during the neonatal period.

In 2007, the World Health Organization (WHO) suggested [15], for regions where <90% of households have access to iodized salt, a daily dose of iodine supplementation of 250 µg in pregnant or lactating women, a daily dose of 150 µg in women of reproductive age—15–49 years—and a daily dose of 90 µg in children below two years of age. Infants’ requirements have been calculated roughly on the average iodine intake of a healthy baby fed on human milk. Considering that the average iodine concentration in breast milk appears to be about 150–180 µg/L in iodine-sufficient areas, the daily iodine production by a mother during lactation has been estimated at about 75–180 µg/day [1]. Children within six months of age should only receive iodine through breast milk from well-supplemented lactating mothers, or through formula milk with adequate iodine content. Children aged seven to 24 months should rather receive supplements as indicated, but only if complementary iodine-fortified food is not available. The daily intake for school-age children is 120 µg [16]. The daily iodine intake, beyond which no health benefit is expected, is 500 µg for pregnant or lactating women (but the safe upper limit for chronic iodine ingestion is not well defined [17]) and 180 µg for children of less than two years age. Theoretically, iodine levels that are greater than those listed above could impair thyroid function. Newborns are particularly at risk as their gland is immature, and, hence, unable to reduce the iodine uptake from blood as a response to excessive exogenous iodine intake (Wolff-Chaikoff effect). This last factor explains why transient hypothyroidism or transient increased TSH in the newborn can be a consequence of maternal iodine overload (e.g., after the use of povidone iodine) especially in areas of moderate deficiency.

## 4. Iodine Deficiency in the Newborn

Studies conducted on healthy preterm and full-term newborns lead to believe that the iodine intakes required to maintain a positive balance are 15 µg/kg/day in full-term newborns and 30 µg/kg/day and up to 60 µg/kg/day in preterm babies [14]. In fact, as far as preterm babies are concerned, iodine deficiency could exacerbate transient hypothyroxinaemia and neither parenteral nutrition nor formula milk completely fulfill the iodine requirements. Nevertheless, there is no real evidence that iodine supplementation decrease morbidity or mortality in preterm babies [18].

Many experts do not recommend routine iodine supplementation in premature babies fed on parenteral nutrition. This is because these babies already receive enough iodine by cutaneous medicaments (disinfectants) or other adventitious sources of iodine (e.g., IV infusions) [19]. The Committee on Clinical Practice Issues of the American Society of Clinical Nutrition recommended, for the preterm infant, parenteral intakes of iodine of 1 µg/kg/day even though this is far below their requirements [20,21].

However, in recent years many efforts have been made towards a “free from iodine excess” environment in Obstetrics and Neonatology Units and, for this reason, the “environmental” iodine should not be considered anymore as a valuable source.

A research published on the Cochrane Database in 2006 [18] on randomized or quasi-randomized controlled trials—compares a policy of supplementing enteral or parenteral feeding with iodine (more than 30 µg/kg/day) *vs.* placebo or no supplementation in preterm infants, concluding that there were no sufficient data to determine whether iodine supplementation could reduce mortality or morbidity in preterm babies. No clear data has shown as yet the benefits of prophylactic thyroid hormones in preterm infants, in order to reduce neonatal mortality, neonatal morbidity, or improve neurodevelopment outcomes so far [22]; a small number of infants enrolled in the tests that document this review does not allow to detect moderate, yet clinically important differences on the effects of thyroid hormone therapy in preterm infants. Preterm babies born before 30 weeks of gestation are prone to transient hypothyroxinaemia. If hypothyroxinemia appears within a crucial period for brain development, preterm babies require particular care in terms of iodine supplementation in order to mimic, as much as possible, intrauterine hormone environment [23,24].

The cause of hypothyroxinaemia in preterm babies is not fully clear. Iodine deficiency contributes to about 30% of cases in enterally fed preterm infants within 27 to 30 weeks of gestation, however, this contribution can be greater in younger babies who have a very low parenteral iodine supply [23].

According to literature, an enteral intake of at least 30–40 µg iodine/kg/day is required in order to achieve a positive iodine balance in healthy preterm infants. The ESPGHAN (*European Society for Paediatric Gastroenterology, Hepatology and Nutrition*) Committee on Nutrition, for example, recommends from 11 to 55 µg/kg/day [25]. As the iodine content of preterm formula varies from about 7 to 25 μg/dL (Table 3), the formula iodine content will not always meet the suggested intakes [26].

It is important to remember that iodine content in milk from mothers of preterm babies seems to be lower than that of mothers of term babies (100–150 µg/L *vs.* 150–180 µg/L). Adding fortifiers to maternal milk raises iodine content to 20–25 μg/dL. The current standard regimes of parenteral nutrition ensure an iodine intake of about 3 µg/kg/day (at 150 mL/kg/day), thus, most infants suffer a negative iodine balance in the first weeks of life.

**Table 3:** Some of commonly used Preterm Infant Formulas: iodine content.

Milk	Iodine (µg/100 mL)
Plasmon 0	25
Pre Aptamil	25
Aptamil PDF	20
Mellin 0 Post	20
Pre Nidina	17.3
Humana 0B	15
Miltina 0	15
Pre Humana	12
Formulat 0	7.4

## 5. Conclusions

Iodine deficiency can be defined as the world’s greatest single cause of preventable brain damage. Important in the synthesis of thyroid hormones, iodine plays a major role in the early growth and development of many organs, particularly the brain and the central nervous system, and in many metabolic pathways. Consequently iodine insufficiency occurring during pregnancy and early infancy, if the maternal/fetal or neonatal thyroid is no longer able to produce enough thyroid hormones, can impair neuro-cognitive development.

Urinary iodine (UI) is a sensible marker of recent iodine intake but it is not reliable for individual diagnosis and treatment as it would be necessary to collect repeated urine samples from an individual over a period of time in order to evaluate its iodine status. Goiter rate, serum TSH, or Tg levels as well are used to assess the iodine status in a population, while thyroid hormones measurement remains a fairly unprecise methodology for the detection of iodine deficiency.

Further research will be useful for better defining methods of assessment of iodine intake and iodine status [27]. Prevention of fetal and neonatal hypothyroidism, caused by iodine deficiency, starts prior to conception and then continues during pregnancy and lactation ensuring an adequate iodine supplementation. Studies on healthy preterm and full-term newborns lead to believe that the iodine intake required to maintain a positive balance are 15 µg/kg/day in full-term newborns and 30 µg/kg/day, and up to 60 µg/kg/day, in preterm babies. In the term newborn, an adequate iodine intake is granted from the first days of age if he or she is formula fed or breastfed by an adequately supplemented mother. Extremely low birth-weight preterm babies (newborns with a birth weight below 1000 g) are at risk of having a negative iodine balance status in the first weeks of life, exacerbating the hypothyroxinaemia of prematurity. It is important that an adequate iodine intake is provided for these babies from the first days of life, nevertheless, neither parenteral nutrition or formula milk completely fulfill iodine requirements. On the other hand, iodine antiseptics should be avoided during pregnancy or lactation and in the newborn: a “free from iodine excess” environment in Obstetrics and Neonatology Units should be assured as far as possible.

More studies are required in order to clearly demonstrate the real benefits deriving from supplementation of preterm formulas [18,28], as well as further large prospective studies are urgently required to exactly define, not only the short term advantages, but also the long term positive effects (for example, on mental development) [29,30] so that a universally accepted iodine supplementation plan in preterm babies can be put in place.
